# Clinical effects of novel susceptibility genes for beta-amyloid: a gene-based association study in the Korean population

**DOI:** 10.3389/fnagi.2023.1278998

**Published:** 2023-10-12

**Authors:** Bo-Hyun Kim, HyunWoo Lee, Hongki Ham, Hee Jin Kim, Hyemin Jang, Jun Pyo Kim, Yu Hyun Park, Mansu Kim, Sang Won Seo

**Affiliations:** ^1^Alzheimer's Disease Convergence Research Center, Samsung Medical Center, Seoul, Republic of Korea; ^2^Department of Health Sciences and Technology, SAIHST, Sungkyunkwan University, Seoul, Republic of Korea; ^3^Neuroscience Center, Samsung Medical Center, Seoul, Republic of Korea; ^4^Department of Neurology, Samsung Medical Center, Sungkyunkwan University School of Medicine, Seoul, Republic of Korea; ^5^Department of Neurology, Seoul National University Hospital, Seoul, Republic of Korea; ^6^Artificial Intelligence Graduate School, Gwangju Institute of Science and Technology, Gwangju, Republic of Korea

**Keywords:** Alzheimer’s disease, PET, GWAS, amyloid-beta (Abeta), gene

## Abstract

Amyloid-beta (Aβ) is a pathological hallmark of Alzheimer’s disease (AD). We aimed to identify genes related to Aβ uptake in the Korean population and investigate the effects of these novel genes on clinical outcomes, including neurodegeneration and cognitive impairments. We recruited a total of 759 Korean participants who underwent neuropsychological tests, brain magnetic resonance imaging, ^18^F-flutemetamol positron emission tomography, and microarray genotyping data. We performed gene-based association analysis, and also performed expression quantitative trait loci and network analysis. In genome-wide association studies, no single nucleotide polymorphism (SNP) passed the genome-wide significance threshold. In gene-based association analysis, six genes (*LCMT1*, *SCRN2*, *LRRC46*, *MRPL10*, *SP6*, and *OSBPL7*) were significantly associated with Aβ standardised uptake value ratio in the brain. The three most significant SNPs (rs4787307, rs9903904, and rs11079797) on these genes are associated with the regulation of the *LCMT1*, *OSBPL7*, and *SCRN2* genes, respectively. These SNPs are involved in decreasing hippocampal volume and cognitive scores by mediating Aβ uptake. The 19 enriched gene sets identified by pathway analysis included axon and chemokine activity. Our findings suggest novel susceptibility genes associated with the uptake of Aβ, which in turn leads to worse clinical outcomes. Our findings might lead to the discovery of new AD treatment targets.

## Introduction

Aging population is growing worldwide. By 2050, the world’s population with age over 65 is anticipated to increase to approximately 1.5 billion (United Nations Department of Economic and Social Affairs, 2021). WHO reported in 2012 that about 150 million people would be impacted by dementia by 2050 (Patterson, 2018), which would subsequently increase the total costs of Alzheimer’s disease (AD), the most common cause of dementia, to over $1 trillion (Prince et al., 2015). In South Korea, the number of patients with dementia in 2021 was estimated at 910,726 and the prevalence rate over 86 years old was estimated at 36.66%, but it is anticipated to exceed 3 million by 2050 (Ministry of Health and Welfare, 2020). Therefore, early diagnosis and intervention are critical to reduce all the burdens that dementia causes.

AD is caused by the accumulation of β-amyloid (Aβ) plaques and neurofibrillary tangles, with subsequent neurodegeneration and cognitive decline (Serrano-Pozo et al., 2011). The heritability of AD is reported to be approximately 60–80% (Gatz et al., 2006). AD also has a complex genetic aetiology. In addition to apolipoprotein E (*APOE*), a well-known risk factor for AD, previous large-scale genome-wide association studies (GWAS) have identified more than 20 genes for late-onset AD (LOAD) (Jun, 2010; Lambert et al., 2013; Wang et al., 2016). However, since their discovery, AD-related genetic variants could account for only about 30% of the clinical diagnosis (phenotype) of AD (Adams et al., 2016). Recent studies have focused on AD endophenotypes, including Aβ and neurodegeneration markers. This approach related to endophenotypes may help identify additional AD-related genetic variants (Potkin et al., 2009; Ramanan et al., 2015; Maxwell et al., 2018; Elsheikh et al., 2020; Homann et al., 2022).

With the development of Aβ positron emission tomography (PET), there is increasing evidence showing the relationships between Aβ uptake and genetic variations (Ramanan et al., 2014, 2015; Li et al., 2015; Apostolova et al., 2018; Raghavan et al., 2020; Kim H. R. et al., 2021). Previous studies based on non-Hispanic whites (NHWs) identified novel genetic variants associated with Aβ uptake in the AD brain (Ramanan et al., 2014, 2015; Li et al., 2015; Apostolova et al., 2018; Raghavan et al., 2020). The latest studies have reported differences in the frequency of Aβ positivity according to various ethnicities (Deters et al., 2021; Kim J. et al., 2021; Wilkins et al., 2022); compared to NHWs, African Americans and Asians had a lower frequency of Aβ positivity. As a result, investigations on the relationships between Aβ uptake and genetic variations in different populations are necessary. However, it is difficult to examine the previously identify genetic variants in Asians, and genetic association studies related to Asians are still insufficient.

Recent genetic association studies have increased our understanding of the underlying genetic architecture of AD. However, identified genetic factors only account for a small fraction of heritability (Maher, 2008; Manolio et al., 2009; Adams et al., 2016). Missing heritability results from the lower explanation power of each causal variant that did not meet the GWAS’s strict statistical threshold or/and the incomplete linkage disequilibrium (LD) between causal and genotyped variants (Yang et al., 2010). However, the gene-based analysis considers genes as units of association, and this analysis may be a valuable complement to GWAS. This analysis may be useful in identifying novel genes associated with traits by reducing multiple comparison corrections. Additionally, this analysis might be helpful to replicate findings, particularly for genetic variants with allelic heterogeneity between populations (Yang et al., 2010; Hong et al., 2012).

**Figure 1:**
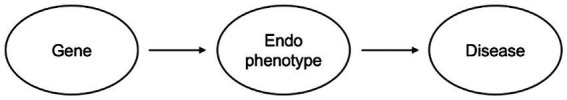
A conceptual diagram of endophenotype conceptual analysis envisioned by Gottesman and Gould (2003). The possible relationship between gene, endophenotype, and disease. Endophenotypes lies along the pathway from gene to disease.

Because the ethnic differences in frequency of Aβ and genetic variants have been reported, more genetic studies trying to identify genetic variants associated with AD in the Asian population are needed. The objective of study is to identify novel genes associated with Aβ uptake in the Korean population. In the present study, we used Aβ uptake as an endophenotype of AD that lies along the pathway from genes to disease (Figure 1) and hypothesised that genes associated with Aβ uptake might affect the development of AD. The endophenotype conceptual analysis and gene-based analysis may help to increase statistical power and facilitate the biological interpretation of results. By adopting these strategies, we expected to identify novel associations between genes and Aβ uptake in the Korean population. Firstly, we performed genetic association analysis on a Korean population-based AD sample. Secondly, we also conducted expression quantitative trait loci (eQTL) analysis to identify the functional role of genetic variants. Furthermore, we investigated the relationships between these SNPs and clinical outcomes. Finally, we performed pathway enrichment and network analysis with gene expression levels in the brain tissue to identify potential AD-related genes.

## Materials and methods

### Study participants

The 759 participants were enrolled from the Korea-Registries to Overcome and Accelerate Dementia research project (K-ROAD). The K-ROAD aims to develop a genotype–phenotype cohort to accelerate the development of novel diagnostic and therapeutic techniques for Alzheimer’s and concomitant cerebrovascular diseases. Nationwide, 25 university-affiliated hospitals in South Korea are participating in the K-ROAD. All participants underwent neuropsychological tests, brain magnetic resonance imaging (MRI), Aβ PET (^18^F-flutemetamol (FMM)), *APOE* genotyping, and microarray genotyping data. The 759 participants consist of Alzheimer’s disease (AD; *n* = 246), amnestic mild cognitive impairment (aMCI; *n* = 255), and cognitively unimpaired (CU) individuals (*n* = 258). All participants with CU met the following criteria: (1) no medical history that was likely to affect cognitive function based on Christensen’s health screening criteria; (2) no objective cognitive impairment in any cognitive domain on a comprehensive neuropsychological test battery (at least −1.0 SD above age-adjusted norms on any cognitive test); and (3) independence in daily living activities. All participants with MCI met the criteria for MCI with the following modifications (Albert et al., 2011); (1) subjective cognitive complaints by the participants or caregivers; (2) objective memory impairment below −1·0 SD on verbal or visual memory tests; (3) no significant impairment in daily living activities; and (4) non-demented status. Participants with dementia met the core clinical criteria of probable AD dementia proposed by the National Institute on Aging-Alzheimer’s Association (NIA-AA; McKhann et al., 2011).

Participants with significant white matter hyperintensities (cap or band >10 mm and longest diameter of deep white matter lesion >25 mm), structural lesions, including cerebral infarction, intracranial haemorrhage, brain tumours, and hydrocephalus on MRI, and abnormal laboratory results on complete blood count, electrolyte, vitamin B12, and folate levels, syphilis serology, and liver, kidney, or thyroid function tests were excluded from the study.

The institutional review board of the Samsung Medical Center approved this study. Written informed consent was obtained from all participants.

### Genotyping and imputation

Genotyping was performed using the Illumina Asian Screening Array BeadChip (Illumina, CA, United States). The quality control (QC) procedures were conducted using PLINK software, and samples that did not satisfy the following criteria were excluded: call rate < 95%, sex-mismatch, excess heterozygosity rate (5 standard deviation from the mean), and identify-by-descent ≥0.125. Additionally, individual markers with the following criteria were excluded: call rate < 98%, minor allele frequency (MAF) < 1%, Hardy–Weinberg equilibrium (HWE) *p* < 10^−6^. Following QC, un-genotyped markers were imputed using Minimac4 and reference haplotypes from HRC-r1.1 on the University of Michigan Imputation Server (Das et al., 2016). Furthermore, post-imputation QC was performed using the following criteria: poor imputation, *r*^2^ ≤ 0.8 and MAF < 1%. Finally, bi-allelic 4,906,407 SNPs in autosomal chromosomes (sex chromosome, mitochondrial, and pseudo autosomal SNPs were excluded) were used for the following analyses.

### Aβ PET acquisition

All participants underwent Aβ PET (^18^F-flutemetamol PET) scans using a Discovery STe PET/CT scanner (GE Medical Systems, Milwaukee, WI, United States). For ^18^F-flutemetamol PET, a 20-min emission PET scan in dynamic mode (consisting of 4 × 5 min frames) was performed 90 min after an injection of a mean dose of 311.5 MBq ^18^F-florbetaben or 197.7 MBq ^18^F-flutemetamol. Three-dimensional PET images were reconstructed in a 128 × 128 × 48 matrix with 2 mm × 2 mm × 3·27 mm voxel size using the ordered-subsets expectation maximisation algorithm (iteration = 4 and subset = 20).

For image processing, we used SPM8^1^ running on MATLAB.^2^ T1-weighted images were corrected for nonuniformity (Sled et al., 1998) and normalised to standard space using a linear transformation. PET images were co-registered, and a Gaussian kernel with an 8 mm full width at half maximum was used for spatial smoothing. Using the whole cerebellum as a reference region, the standardised uptake value ratio (SUVR) images were calculated. The mask of the reference region was obtained from the Global Alzheimer’s Association Information Network (GAAIN) websites^3^ (Klunk et al., 2015). The mean SUVR of cerebral cortical areas was determined using the masks of the standard cortical target region (Klunk et al., 2015) downloaded from GAAIN.

### MRI acquisition and measurement of hippocampal volume

We acquired standardised three-dimensional T1 Turbo Field Echo and three-dimensional fluid-attenuated inversion recovery (FLAIR) images using a 3.0 T MRI scanner (Philips 3.0 T Achieva; Philips Healthcare, Andover, MA, United States), as previously described (Kang et al., 2021).

Images were processed using the CIVET anatomical pipeline (version 2.1.0). The native MRI images were registered to the MNI-152 template by a linear transformation and corrected for intensity nonuniformities using the N3 algorithm (Sled et al., 1998). We used an automated hippocampus segmentation method described in an earlier study that combined a graph cut algorithm with atlas-based segmentation and morphological opening to measure hippocampal volume (Kwak et al., 2013).

### Gene-based association analysis

To assess the effect of genes on Aβ SUVR, a gene-based association analysis was performed by combining the SNP-based *p*-values from a GWAS, which was performed using the PLINK software (Purcell et al., 2007). The statistical analysis was performed on 759 participants from three diagnostic groups (CU, aMCI, and AD) and allelic effects for the Aβ SUVR were measured using an additive model with age, sex, and APOE ε4 counts as covariates. Gene-based association analysis was conducted using the extended Simes procedure (GATES; Li et al., 2011). SNPs that mapped within 20 kb of the 3′ and 5′ untranslated regions were considered “within” the genes. The linkage disequilibrium (LD) was calculated using data for East Asian ancestry from the 1,000 Genomes Project, and SNPs with *r*^2^ < 0.005 were ignored in gene annotation.

### EQTL analysis

We performed an eQTL analysis to determine the functional effect of genetic variants on gene expression. Genotype data and exon-specific expression in the hippocampus, frontal, and temporal cortex were downloaded from the Braineac database^4^ (Ramasamy et al., 2014). The eQTL analysis was performed with the most significant SNPs mapped to identified genes from gene-based association analysis. Multiple comparison correction for the number of tissues was performed, and eQTLs with *q* < 0.05 were considered significant.

### Association analysis between SNPs with clinical outcomes

We hypothesised that the identified genetic variants were related to biomarkers that indicate the severity of disease symptoms. The effects of the most significant SNPs mapped to identified genes on cognitive scores [Mini-Mental State Exam (MMSE) and Clinical Dementia Rating Sum of Boxes (CDR-SB)] and hippocampal volume were investigated. After applying inverse normal transformations to cognitive scores for data normality, associations were tested, and age, sex, education, and ICV volume were used as covariates, if appropriate. In addition, the *mediation package* in R was used for mediation analysis in order to identify the SUVR-mediated pathway from SNP to AD biomarkers (Imai et al., 2010). The genotypes were defined as independent variables, the SUVR as a mediator variable, and the clinical outcomes as continuous dependent variables. The *mediate() function* generated the direct and indirect effects as well as paths from the mediator variable to dependent variables by linear regression.

### Pathway analysis

We performed pathway analysis using GSA-SNP (Nam et al., 2010) to identify functional gene sets associated with Aβ SUVR. The pathway annotations from the Gene Ontology (GO) resource^5^ were downloaded, and gene sets containing 5–200 genes were used for analysis. The summary statistics of GWAS were used, and SNPs that fell within 20 kb of the boundary of a gene were annotated to the gene on the human genome (hg19) coordinate. Gene sets were evaluated by Z-statistics for the identification of enriched gene sets, with gene sets having *q* < 0.05 considered significant.

### Network analysis

We generated a protein–protein interaction network using the WEB-based gene set analysis toolkit (Liao et al., 2019) with the human PPI data from the Biological General Repository for Interaction Datasets. For the seed genes identified in gene-based association analysis, random walk analysis was performed to expand the network. The network was expanded by ranking genes based on their network proximity with the seed genes, and the resulting network consisted of the seed genes and the top 50 neighbouring genes. Functional module detection was performed using HumanBase (Greene et al., 2015) to find the functional gene cluster in human brain tissue. Genes were clustered into the functional modules with tissue-specific networks based on the shared k-nearest nearest-neighbours (SKNN) and the Louvain community-finding algorithm. Functional enrichment analysis was performed for the resulting functional modules using Go terms. Statistical significance was tested by a one-sided Fisher’s exact test and Go terms with *q* < 0.05 were considered significant.

## Results

### Study participants

The demographics and genotypic characteristics of the final samples are listed in Table 1. The age (mean [±standard deviation]) of the participants was 70.6 (±8.5) years; the proportions of female and Aβ + participants were 58.6 and 49.4%, respectively. The proportions of apolipoprotein ε4 carriers were 38.1%.

**Table 1:** Demographics of study participants.

	Total	CU	aMCI	AD
*N*	759	258	255	246
Age, mean (SD)	70.6 (8.5)	68.8 (7.78)	70.6 (8.04)	71.4 (9.46)
Gender, female, *N* (%)	314 (58.6%)	149 (57.8%)	133 (52.2%)	163 (66.3%)
*APOE* ε4 count (0/1/2)	470/228/61	195/58/5	163/73/19	112/97/37
Aβ positivity, *N* (%)	375 (49.4%)	37 (14.3%)	132 (51.8%)	208 (84.6%)
Aβ SUVR, mean (SD)	1.29 (0.34)	1.05 (0.16)	1.28 (0.32)	1.57 (0.3)

The APOE ε4 count represents the number of ε4 copies in rs429358 and rs7412 single nucleotide polymorphisms (SNPs). CU, cognitively unimpaired; aMCI, amnestic mild cognitive impairment; AD, Alzheimer’s disease; Aβ, amyloid beta; SUVR, standardised uptake value ratio.

### GWAS

The considerable association of *APOE4* with Aβ SUVR led us to conduct GWAS with *APOE* ε4 counts as a covariate, where no SNP passed the genome-wide significance threshold of 5 × 10^−8^ (Supplementary Figure S1). Ten SNPs on chromosomes 13, 16, 17, and 21 showed genome-wide suggestive significance (*p* < 1 × 10^−6^) and there is no genomic inflation (*λ* = 1.0).

### Gene-based association analysis

**Figure 2:**
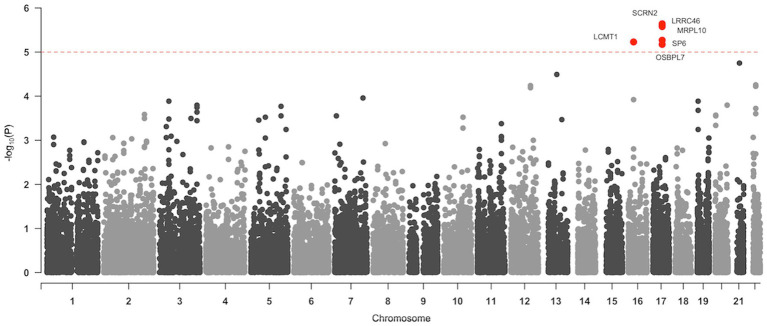
Manhattan plot of gene-based association analysis. The horizontal axis (x-axis) shows the start position of genes on chromosomes, and the vertical axis (y-axis) shows the observed −log_10_ (value of *p*). The red horizontal line indicates a genome-wide significant threshold (value of *p* <1 × 10^−5^, which approximately corresponds to a threshold of the false discovery rate (FDR) corrected value of *p* <0.05). Genes with FDR-corrected value of *p* < 0.05 are coloured in red.

In gene-based association analysis, 3,936,415 SNPs mapped to 26,443 genes on the human genome. One gene on chromosome 16, *LCMT1* (Leucine Carboxyl Methyltransferase 1), and five genes on chromosome 17, *SCRN2* (Secernin 2), *LRRC46* (Leucine Rich Repeat Containing 46), *MRPL10* (Mitochondrial Ribosomal Protein L10), *SP6* (Sp6 transcription factor), and *OSBPL7* (Oxysterol Binding Protein Like 7), were significantly associated with the Aβ SUVR (Figure 2). *SCRN2* on chromosome 17 showed the strongest associations with Aβ SUVR (*q* = 2.33 × 10^−2^). Four additional genes, *LRRC46*, *MRPL10, SP6*, and *OSBPL7*, close to *SCRN2*, were found to be related to Aβ SUVR (*p* = 2.46 × 10^−6^, *p* = 2.64 × 10^−6^, *p* = 5.39 × 10^−6^, *p* = 6.71 × 10^−6^, respectively). Furthermore, the *LCMT1* gene on chromosome 16 was observed to be significantly related to Aβ SUVR (*p* = 5.90 × 10^−6^).

**Figure 3:**
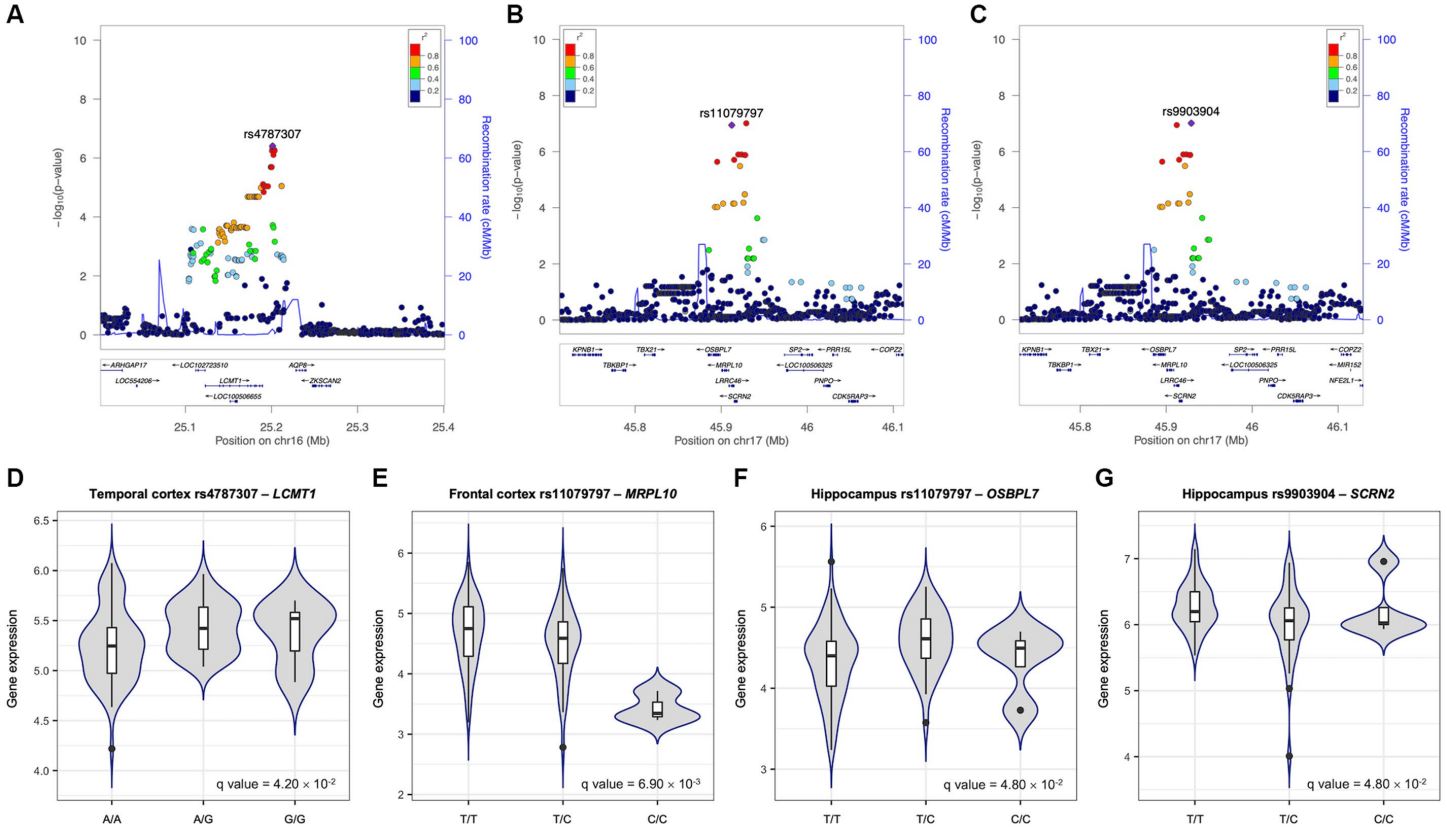
Effects of the most significant SNPs on Aβ SUVR and gene expression. Regional association plots for Aβ standardised uptake value ratio (SUVR) **(A–C)**. All single nucleotide polymorphisms (SNPs) within 200 kb upstream and downstream of rs4787307 on chromosome 16 **(A)**, rs11079797 **(B)**, and rs9903904 **(C)** on chromosome 17 are plotted based on their GWAS −log_10_ (value of *p*s). The most significant SNPs mapped to identified genes in gene-based association analysis are highlighted in violet. The colour scale of the squared correlation (*r*^2^) value is used to label SNPs based on their degree of linkage disequilibrium with the highlighted SNPs. Genes in the region are labelled with arrows denoting the 5′-3′ orientation. All plots are adapted from LocusZoom results. Expression quantitative trait loci (eQTL) plots of association between rs4787307 and the expression levels of *LCMT1* **(D)**, rs11079797 and *MRPL10* **(E)** and *OSBPL7* **(F)**, and rs9903904 and *SCRN2* **(G)**. The x-axes are the genotype of SNP and y-axes are exon-specific gene expression obtained from United Kingdom brain expression consortium (UKBEC).

The genetic effects of the most significant SNPs mapped to the identified six genes are shown in Figures 3A–C. The rs4787307 on the *LCMT1* gene has a risk effect on Aβ SUVR (*β* = 0.258, *p* = 3.91 × 10^−7^), which means that participants with minor alleles have a higher Aβ SUVR than those without minor alleles. The rs9903904 mapped to *SCRN2*, *LRRC46*, and *SP6* genes, and the rs11079797 mapped to *MRPL10* and *OSBPL7* also have risk effects (*β* = 0.655, *p* = 9.73 × 10^−8^ and *β* = 0.623, *p* = 1.14 × 10^−7^, respectively).

### EQTL analysis

To identify the functional effect of genetic variants on gene expression, cis-eQTL analysis was performed. We performed eQTL analysis with three SNPs (rs4787307, rs9903904, and rs11079797) as well as the expression levels of the mapped genes in the frontal and temporal cortex and hippocampus (Figures 3D–G). Our findings indicated that rs4787307 on chromosome 16 significantly regulates the expression of *LCMT1* in the temporal cortex (*q* = 4.20 × 10^−2^), while rs11079797 on chromosome 17 significantly regulates the expression of *MRPL10* (*q* = 6.90 × 10^−3^) and *OSBPL7* (*q* = 4.80 × 10^−2^) in the frontal cortex and hippocampus. In addition, rs9903904 on chromosome 17 influences the expression of *SCRN2* in the hippocampus (*q* = 4.80 × 10^−2^).

### Relationships between SNPs and clinical outcomes

We tested the association between the most significant SNPs on six genes and cognitive scores and hippocampal volume (Table 2). The association tests are shown in Table 2. The rs4787307 in *LCMT1* was significantly associated with CDR-SB (*β =* 0.129, *p* = 2.25 × 10^−2^), left (*β =* −93.19, *p* = 6.39 × 10^−3^) and right hippocampal volume decline (*β =* −106.51, *p* = 1.00 × 10^−2^). The rs11079797 in *MRPL10* and *OSBPL7* was significantly associated with MMSE (*β =* −0.372, *p* = 2.61 × 10^−3^), left (*β =* −181.2, *p* = 1.90 × 10^−2^) and right hippocampal volume decline (*β =* −164.56, *p* = 3.83 × 10^−2^). In addition, rs9903904 in *SCRN2*, *LRRC46*, and *SP6* was significantly associated with MMSE (*β =* −0.453, *p* = 4.74 × 10^−4^), CDR-SB (*β =* 0.321, *p* = 1.48 × 10^−2^), left (*β =* −213.2, *p* = 8.72 × 10^−3^), and right hippocampal volume (*β =* −203.45, *p* = 2.34 × 10^−2^).

**Table 2:** Results of associations analysis of SNPs with clinical outcomes.

SNP	CHR	BP	MMSE		CDR-SB		Left hippocampus		Right hippocampus	
			β	*q*	β	*q*	β	*q*	β	*q*
rs4787307	16	25,201,271	−0.11	5.57 × 10^−2^	0.13	**3.00 × 10^−2^**	−93.19	**1.92 × 10^−2^**	−106.51	**1.00 × 10^−2^**
rs11079797	17	45,912,302	−0.34	**1.92 × 10^−2^**	0.24	5.57 × 10^−2^	−181.24	**2.85 × 10^−2^**	−164.56	**3.83 × 10^−2^**
rs9903904	17	45,929,169	−0.42	**1.00 × 10^−2^**	0.32	**2.54 × 10^−2^**	−213.18	**2.09 × 10^−2^**	−203.45	**2.34 × 10^−2^**

Associations with *q* < 0.05 are indicated in bold in the table. CHR, chromosome; BP, position of the SNP; MMSE, mini-mental state exam; CDR-SB, clinical dementia rating sum of boxes; Left hippocampus, left hippocampal volume; Right hippocampus, right hippocampus volume; *q*, *q*-value.

**Figure 4:**
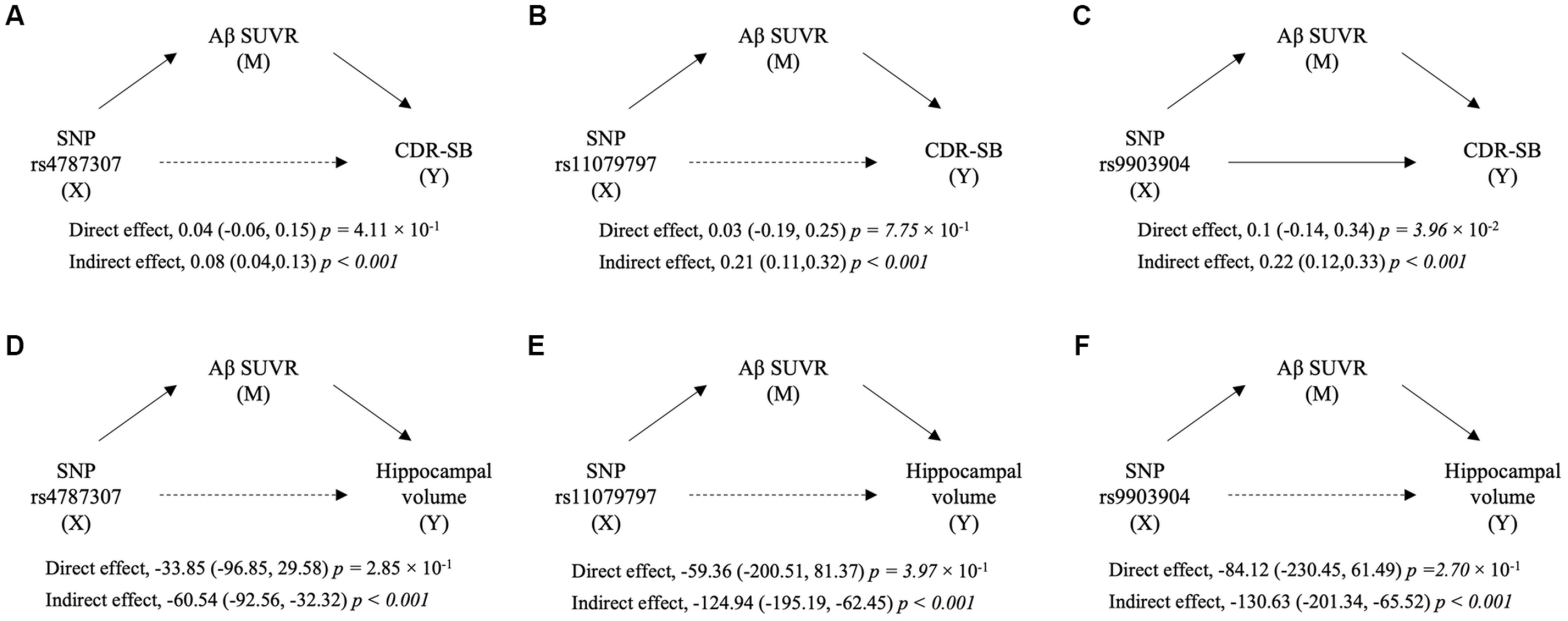
The diagram of mediation analyses for the relation of SNP with cognitive scores and hippocampal volume. The mediation implemented by Aβ standardised uptake value ratio (SUVR) from single nucleotide polymorphisms (SNPs) on Clinical Dementia Rating Sum of Boxes (CDR-SB) **(A–C)** and from SNP on left hippocampal volume **(D–F)**. X, M, and Y indicate predictor, mediator, and outcome variables in mediation analyses, respectively. The direct and indirect effects are presented as coefficients (95% confidence interval) with a value of *p*.

To identify the Aβ uptake-mediated pathways from SNP to clinical outcomes, we performed mediation analysis with SUVR as the mediator variable and clinical outcomes as dependent variables (Figure 4; Supplementary Table S1). Three SNPs had significant indirect effects when CDR-SB was set as the outcome (rs4787307, effect = 0.08, *p* < 0.001; rs11079797, effect = 0.21, *p* < 0.001; and rs9903904, effect = 0.22, *p <* 0.001; respectively), while only rs9903904 had a significant direct effect (effect = 0.1, *p <* 3.65 × 10^−2^). In addition, when left hippocampal volume was set as the outcome, we found significant indirect effects of three SNPs (rs4787307, effect = −60.54, *p* < 0.001; rs11079797, effect = −124.94, *p* < 0.001; rs9903904, effect = −130.63, *p* < 0.001; respectively), but the direct effects were not significant. The mediation results for MMSE and right hippocampal volume are presented in Supplementary Table S1. These findings indicate that the SNP could affect hippocampal volume and cognitive scores, primarily through the mediation of Aβ SUVR.

### Pathway analysis

**Figure 5:**
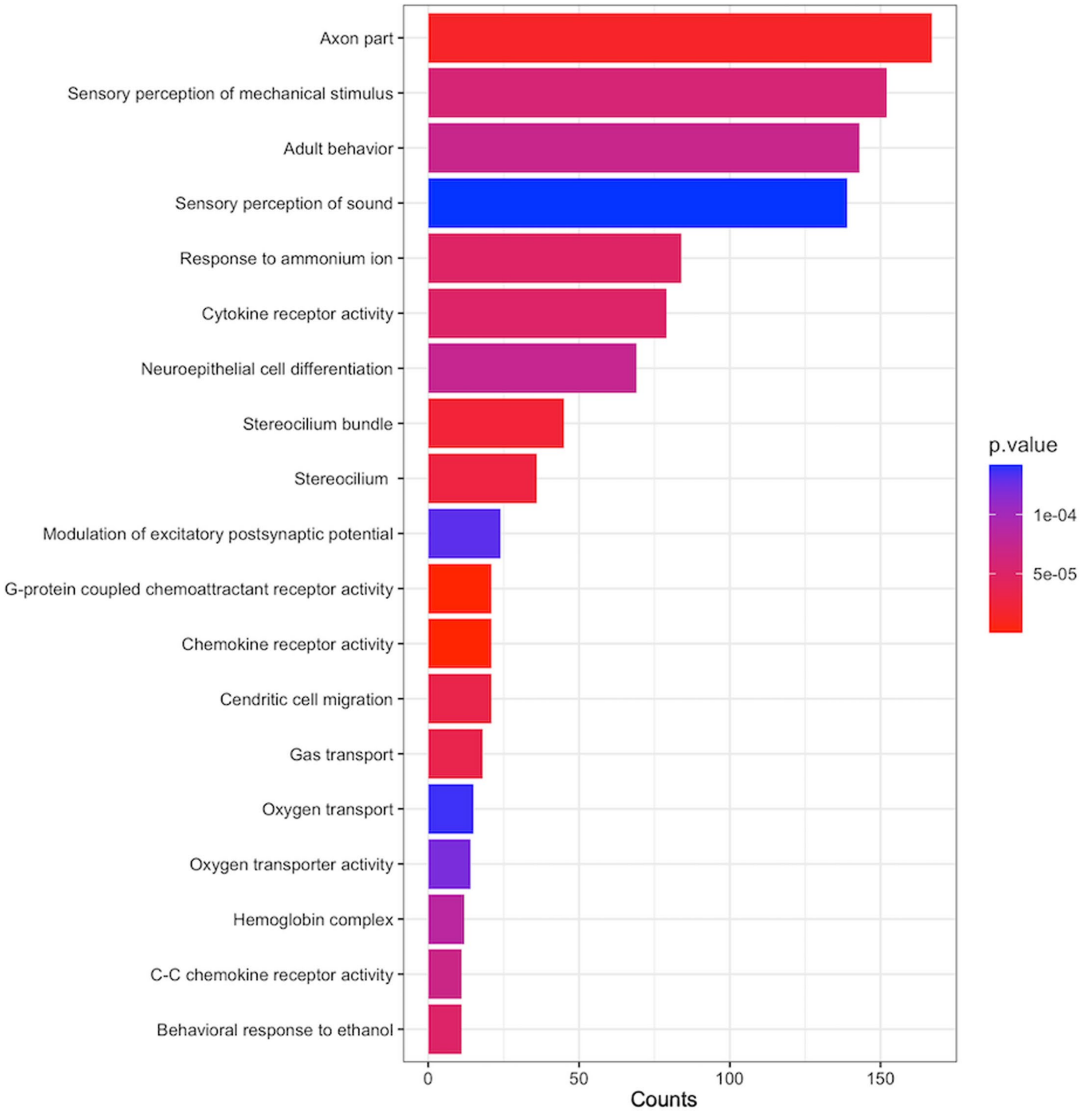
Enriched pathways associated with Aβ SUVR. The y-axis is the name of gene sets from gene ontology (GO), the x-axis ‘count’ indicates the number of genes included in the gene sets, and the colours indicate the pathway analysis *p*-values from GSA-SNP2.

To identify functional gene sets associated with Aβ SUVR, we performed pathway analysis using SNP *p*-values from GWAS. The 6,432 gene sets have been examined, and 19 gene sets were enriched for Aβ SUVR (Figure 5). Gene sets related to axon parts, cytokine, and chemokine activity were enriched. More detailed results and a list of genes contained in the gene set are presented in Supplementary Table S2.

### Network analysis

We constructed a network consisting of six seed genes identified in the gene-based association analysis and 50 top-ranking neighbour genes (Supplementary Figure S2). The 56 genes in the constructed PPI network were clustered into five functional modules in the cerebral cortex. Modules 1, 2, 3, 4, and 5 included 6, 10, 6, 8, and 10 genes, respectively (Supplementary Figure S3; Supplementary Table S3). In enrichment analysis, eight genes clustered into M1 were enriched for DNA repair, recombination-related processes, and dephosphorylation (Table 3). M2 and M4 genes were enriched for cell cycle-related processes, and M5 was enriched for the proteolysis-related process.

**Table 3:** Pathways enriched in functional gene modules.

Module name	GO name	*q*
M1	Regulation of double-strand break repair *via* homologous recombination	1.14 × 10^−3^
	Regulation of DNA recombination	1.47 × 10^−3^
	Regulation of double-strand break repair	1.48 × 10^−3^
	Double-strand break repair *via* homologous recombination	1.97 × 10^−3^
	Recombinational repair	1.97 × 10^−3^
	Regulation of DNA repair	1.97 × 10^−3^
	DNA recombination	2.75 × 10^−3^
	Double-strand break repair	2.75 × 10^−3^
	Regulation of response to DNA damage stimulus	2.75 × 10^−3^
	Protein dephosphorylation	2.75 × 10^−3^
	DNA repair	5.80 × 10^−3^
	Regulation of DNA metabolic process	5.80 × 10^−3^
	Dephosphorylation	5.80 × 10^−3^
M2	Positive regulation of protein catabolic process	2.75 × 10^−3^
	Regulation of protein catabolic process	5.80 × 10^−3^
	Proteasomal protein catabolic process	5.80 × 10^−3^
	Positive regulation of catabolic process	5.95 × 10^−3^
	Positive regulation of MAPK cascade	6.32 × 10^−3^
	Negative regulation of cell cycle	6.99 × 10^−3^
M3	Peptidyl-serine modification	2.75 × 10^−3^
	Peptidyl-serine phosphorylation	2.75 × 10^−3^
M4	Mitotic cell cycle phase transition	3.07 × 10^−3^
	Cell cycle phase transition	3.87 × 10^−3^
M5	Negative regulation of proteolysis	5.83 × 10^−3^
	Regulation of protein catabolic process	7.72 × 10^−3^

M, module; GO, gene ontology; *q*, *q*-value.

## Discussion

In the present study, we performed a genetic association study to identify novel genes associated with Aβ uptake in the brain. Our major findings are as follows. First, six genes, *LCMT1, SCRN2*, *LRRC46*, *MRPL10*, *SP6*, and *OSBPL7*, were associated with Aβ uptake independent of the APOE effects. Second, the most significant SNPs (rs4787307, rs9903904, and rs11079797) mapped to identified genes were predictive of neurodegeneration and cognitive outcomes with the mediation of Aβ uptake. Finally, the genes identified by pathway-based analysis and by combining network and functional enrichment analysis, are related to AD pathophysiology. Overall, our findings indicated that these six genes might be novel potential targets of AD.

Our conclusion that these identified genes are associated with Aβ uptake, which in turn leads to poor clinical outcomes, is supported by the following observations: (1) Six genes (*LCMT1, SCRN2*, *LRRC46*, *MRPL10*, *SP6*, and *OSBPL7*) were associated with Aβ uptake; (2) eQTL analysis revealed that the SNPs in these genes were associated with the regulation of genes; (3) these SNPs were predictive of neurodegeneration and cognitive impairments. Previously, studies reported that these genes were associated with AD pathophysiology. Particularly, *LCMT1* increases PP2A activity, which in turn leads to decreased tau hyperphosphorylation in the AD (Torrent and Ferrer, 2012). A previous study also reported that microglial expression of *LRRC46* in the CA1 region of the hippocampus was significantly different between the AD group and the control group (Mastroeni et al., 2018). Furthermore, the *SCRN2, MRPL10*, and *OSBPL7* are related to proteolysis, mitochondrial translation, and cholesterol metabolism, respectively, which play important roles in AD pathogenesis (Bishop et al., 2010; Strooper, 2010; Swerdlow, 2011; Picard and McEwen, 2014; Xue et al., 2018). In fact, a previous study suggested that genetic variants in *MRLP10* and *OSBL7* are associated with cognitive decline in AD (Sherva et al., 2014). However, to our knowledge, there are no studies showing that these genes influence the pathophysiology of AD through increased Aβ uptake. Notably, in this study, Aβ uptake mediated the relationships between these SNPs and neurodegeneration or between these SNPs and cognitive outcomes. As a result, our findings might help achieve a better understanding of AD pathophysiology and help uncover novel therapeutic targets for AD.

The mechanisms by which these genetic variants are predictive of worse clinical outcomes remain to be elucidated. These findings, however, might be explained by our other findings from pathway-based analysis and the combination of network analysis and functional enrichment. The 19 gene sets identified through pathway-based analysis are associated with axon part, cytokine receptor activity, modulation of excitatory postsynaptic potential, and chemokine receptor activity, which is related to AD pathophysiology (Figure 4; Akiyama et al., 2000; Ardiles et al., 2012; Millecamps and Julien, 2013; Liu et al., 2014; Nagae and Araki, 2016; Sleigh et al., 2019). Additionally, enriched pathways in functional modules identified by a combination of network analysis and functional enrichment revealed that the genes consisting of networks were related to critical AD processes, such as DNA repair and recombination processes, cell cycle-related processes, and dephosphorylation (Table 3; Sheng et al., 1998; Iqbal et al., 2010; Shanbhag et al., 2019). Cell cycle deficits are associated with various neurogenerative disorders, including AD. Several genes and proteins, such as DKs and cyclins, have been linked to cell cycle dysregulation in AD (Yang et al., 2003; Hamdane and Buée, 2007; Zhang et al., 2012), and cell cycle proteins have been reported to be related to tau phosphorylation (Conejero-Goldberg et al., 2008; Seward et al., 2013; Kim et al., 2016).

The strength of our study is the recruitment of participants using a standardised diagnostic protocol, including detailed neuropsychological tests, Aβ PET, and brain MRI. However, the present study had some limitations. We identified novel genes related to AD using gene-based association analysis, but our sample size was moderate for a genetic association study. As a result, the replication of our findings in larger, independent datasets is needed. In addition, we used only the Korean population; further studies in a racially diverse sample are needed to generalise our findings. Nevertheless, the fact that so little research has been conducted on the Asian population makes our current study notable. We have identified genes associated with Aβ uptake whose involvement in Aβ uptake was not reported in previous European studies, highlighting the importance of genetic association studies in a diverse population.

In conclusion, by utilising gene-based association analysis, we identified new APOE-independent associations of six genes, *LCMT1, SCRN2*, *LRRC46*, *MRPL10*, *SP6*, and *OSBPL7*, with Aβ uptake in the Korean cohort. The SNPs on these genes were also related to decreased hippocampal volume and cognitive scores. Furthermore, we identified several functional gene sets that may contribute to AD through pathway enrichment and network analysis. Our findings may contribute to understanding the genetic architecture underlying Aβ uptake and its relationships with Alzheimer’s disease.

## Data availability statement

The original contributions presented in the study are publicly available. This data can be found here: https://www.ebi.ac.uk/eva/?eva-study=PRJEB66172.

## Ethics statement

All procedures performed in studies involving human participants were in accordance with the ethical standards of the institutional and/or national research committee and with the 1964 Helsinki Declaration and its later amendments or comparable ethical standards. Informed consent was obtained from all individual participants included in the study. The Institutional Review Board of Samsung Medical Center approved the study protocol, and all methods were performed according to the approved guidelines. The studies were conducted in accordance with the local legislation and institutional requirements. The participants provided their written informed consent to participate in this study.

## Author contributions

B-HK: Conceptualization, Formal analysis, Methodology, Visualization, Writing – original draft. HL: Formal analysis, Methodology, Writing – original draft. HH: Formal analysis, Methodology, Writing – original draft. HK: Data curation, Writing – review & editing. HJ: Data curation, Writing – review & editing. JK: Data curation, Writing – review & editing. YP: Formal analysis, Methodology, Writing – original draft. MK: Conceptualization, Supervision, Writing – review & editing. SS: Conceptualization, Supervision, Writing – review & editing.

## Footnotes

^1^https://www.fil.ion.ucl.ac.uk/spm/

^2^https://www.mathworks.com/

^3^http://www.GAIN.org

^4^http://www.braineac.org/

^5^http://geneontology.org
