# Supplementary material for: Clinical effects of novel susceptibility genes for beta-amyloid: a gene-based association study in the Korean population
Source: Front Aging Neurosci. 2023 Oct 12;15:1278998. doi: 10.3389/fnagi.2023.1278998 (PMC10602697; doi:10.3389/fnagi.2023.1278998)
Supplement: Supplementary file 1 [file Data_Sheet_1.ZIP › supplementary figures.docx]

**Supplementary Materials**

Proposed title: Clinical effects of novel susceptibility genes for beta-amyloid: A gene-based association study in the Korean population

Authors: Bo-Hyun Kim, HyunWoo Lee, Hongki Ham, Hee Jin Kim, Hyemin Jang, Jun Pyo Kim, Yu Hyun Park, Mansu Kim, Sang Won Seo

**Supplementary Figures**

**
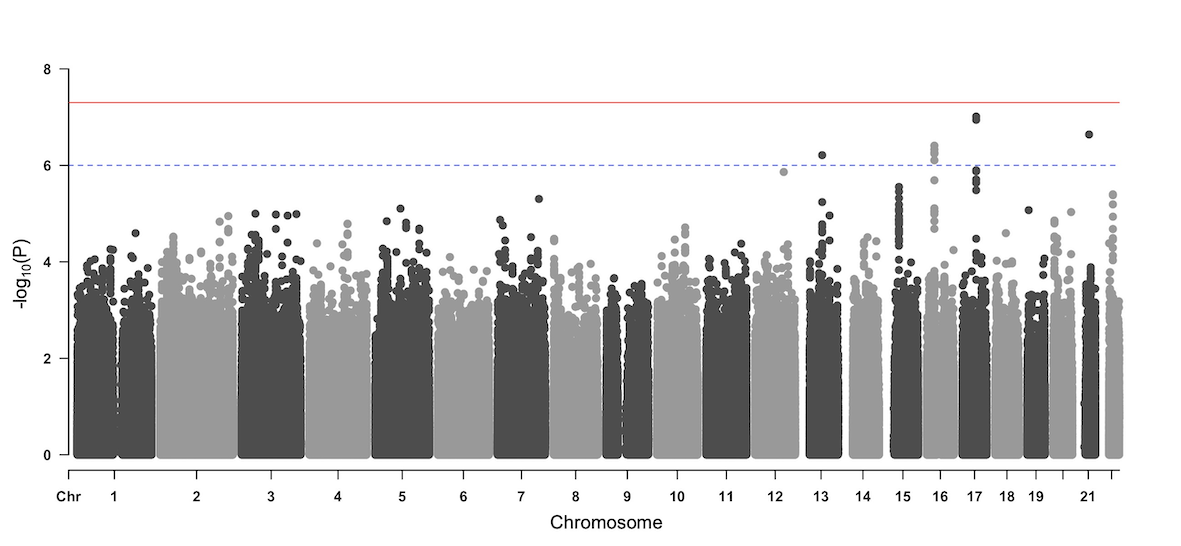
**

**Supplementary Figure S1. Manhattan plot of the GWAS for Aβ SUVR.** The horizontal axis (x-axis) shows the position of SNPs on chromosomes, and the vertical axis (y-axis) shows the observed -log_10_(p-value). The red horizontal line indicates a genome-wide significant threshold (5 × 10^-8^) and the blue horizontal line indicates a suggestive threshold (1 × 10^-6^).


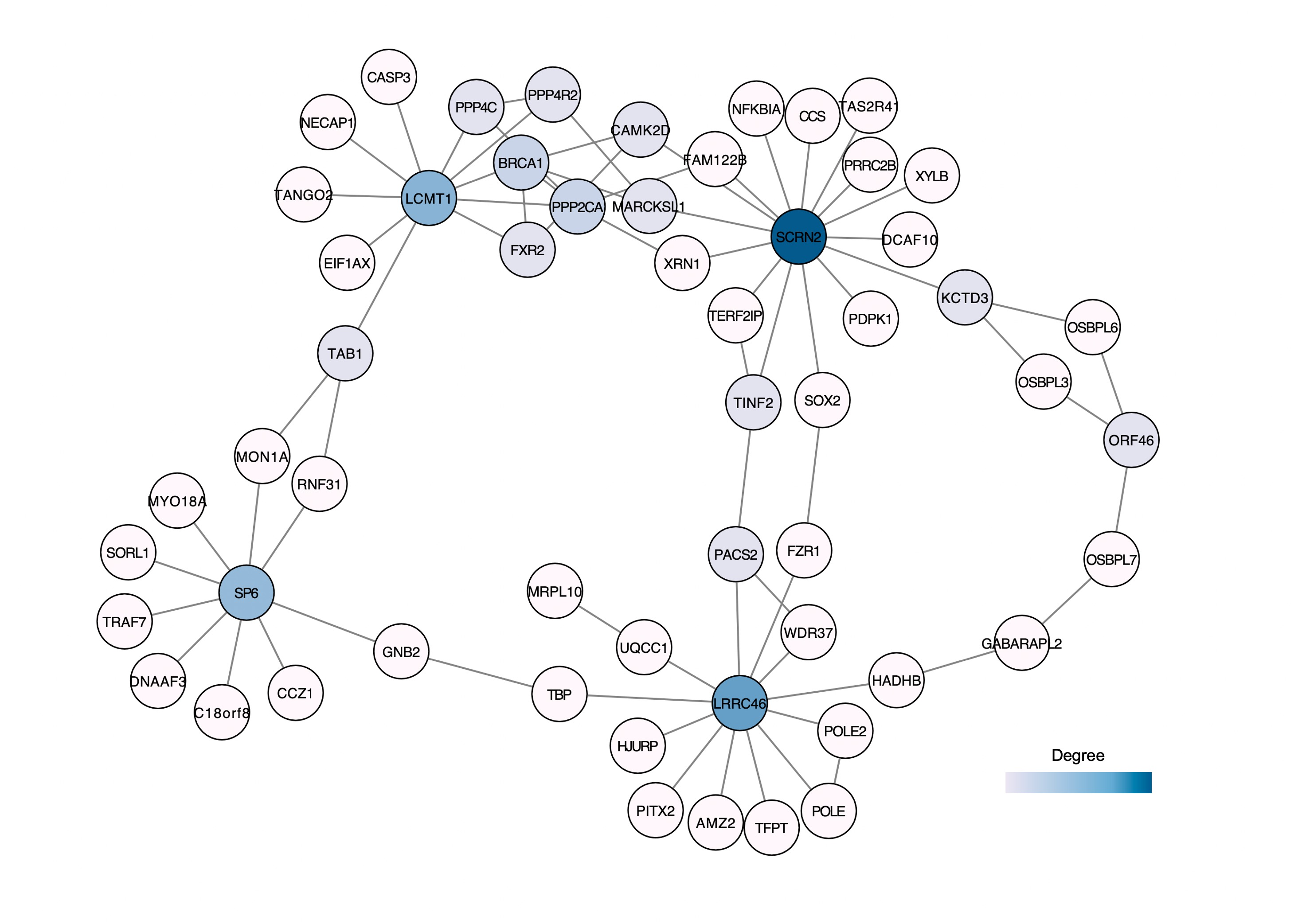


**Supplementary Figure S2. Expanded protein-protein interaction (PPI) network.** The PPI network consists of 6 seed genes and 50 top-ranking neighbour genes.


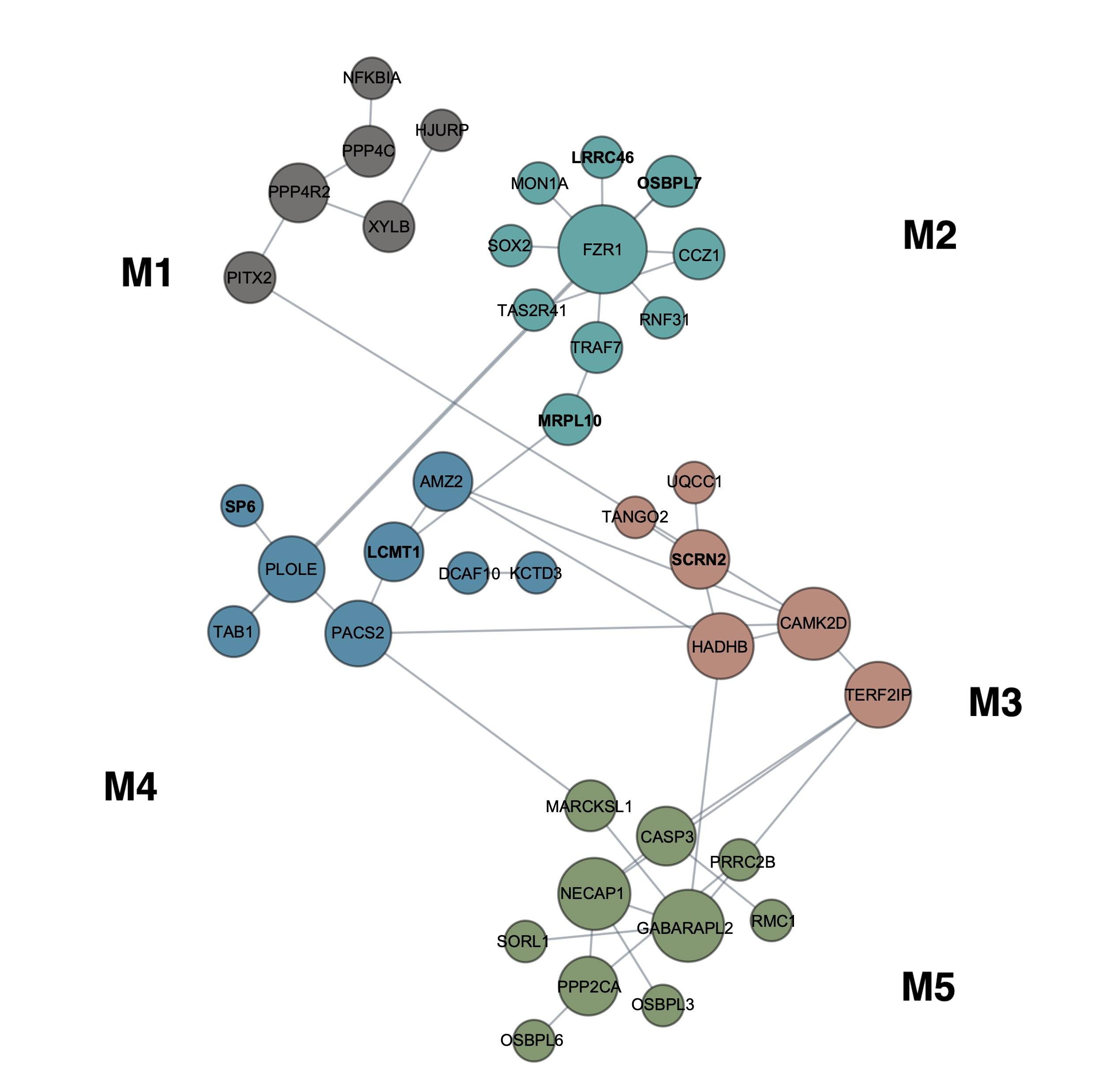


**Supplementary Figure S3. Functional module in the cerebral cortex.** Genes in the PPI network are clustered into five functional modules in cerebral cortex tissue. Genes identified in gene-based association analysis are marked in bold.
